# A genome-wide miRNA screen revealed miR-603 as a MGMT-regulating miRNA in glioblastomas

**DOI:** 10.18632/oncotarget.1974

**Published:** 2014-05-14

**Authors:** Deepa Kushwaha, Valya Ramakrishnan, Kimberly Ng, Tyler Steed, Thien Nguyen, Diahnn Futalan, Johnny C. Akers, Jann Sarkaria, Tao Jiang, Dipanjan Chowdhury, Bob S. Carter, Clark C. Chen

**Affiliations:** ^1^ Dept. of Radiation Oncology, Dana-Farber Cancer Institute, Boston, MA; ^2^ Center for Theoretical and Applied Neuro-Oncology, Moores Cancer Center, Division of Neurosurgery, University of California San Diego, San Diego, CA; ^3^ Mayo Clinic, Department of Radiation Oncology, Rochester, MN; ^4^ Department of Neurosurgery, Tiantan Medical Center, Beijing, China

**Keywords:** microRNA, Glioblastoma, MGMT, Temozolomide, miRNA cooperativity

## Abstract

MGMT expression is a critical determinant for therapeutic resistance to DNA alkylating agents. We previously demonstrated that MGMT expression is post-transcriptionally regulated by miR-181d and other miRNAs. Here, we performed a genome-wide screen to identify MGMT regulating miRNAs. Candidate miRNAs were further tested for inverse correlation with MGMT expression in clinical specimens. We identified 15 candidate miRNAs and characterized the top candidate, miR-603. Transfection of miR-603 suppressed MGMT mRNA/protein expression *in vitro* and *in vivo*; this effect was reversed by transfection with antimiR-603. miR-603 affinity-precipitated with MGMT mRNA and suppressed luciferase activity in an MGMT-3'UTR-luciferase assay, suggesting direct interaction between miR-603 and MGMT 3'UTR. miR-603 transfection enhanced the temozolomide (TMZ) sensitivity of MGMT-expressing glioblastoma cell lines. Importantly, miR-603 mediated MGMT suppression and TMZ resistance were reversed by expression of an MGMT cDNA. In a collection of 74 clinical glioblastoma specimens, both miR-603 and miR-181d levels inversely correlated with MGMT expression. Moreover, a combined index of the two miRNAs better reflected MGMT expression than each individually. These results suggest that MGMT is co-regulated by independent miRNAs. Characterization of these miRNAs should contribute toward strategies for enhancing the efficacy of DNA alkylating agents.

## INTRODUCTION

O^6^-methylguanine methyl transferase (MGMT) encodes an evolutionarily conserved protein, the primary function of which is to repair guanine nucleotides that are alkylated at the O^6^ position [1]. O^6^-methyl guanine constitutes the major cytotoxic lesion induced by DNA alkylating chemotherapeutic agents, including temozolomide [2]. In model organisms spanning the entire evolutionary ladder [3] and in every cell line tested [4], high MGMT expression is associated with cellular resistance to DNA alkylating agents. Clinically, high MGMT mRNA and protein expression have been associated with therapeutic resistance to DNA alkylating agents in a number of cancers [5].

A major mechanism of MGMT regulation involves methylation of CpG islands in the promoter region [6]. Methylation of these regions suppresses MGMT transcription [7, 8]. This mechanism of regulation is particularly important in glioblastoma, the most common form of primary brain cancer [9]. MGMT promoter methylation has been associated with favorable response to temozolomide in three randomized controlled trials, including EORTC-NCIC [10], NOA-8 [11], and the Nordic Trial [12].

While MGMT promoter status has been associated with therapeutic response to temozolomide for glioblastoma patients, there has been reluctance to restrict temozolomide treatment to patients harboring MGMT promoter methylated glioblastomas [13, 14]. One of the major reasons underlying this reluctance is the observation that a significant portion of MGMT promoter unmethylated tumors harbor MGMT levels comparable to those with methylated MGMT promoters [7, 15, 16]. These findings suggest that many glioblastoma patients with unmethylated MGMT can still derive benefit from temozolomide treatment. Thus, understanding the mechanism underlying the heterogeneity of MGMT expression can fundamentally impact clinical care of glioblastoma patients.

Our previous work showed that the low MGMT expression in promoter unmethylated tumors was due in part to the expression of miR-181d, a miRNA that suppresses MGMT expression [17]. However, our analysis indicated that additional miRNAs may be involved in regulating MGMT expression. Here, we identify miR-603 as another MGMT regulating miRNA. We demonstrate that miR-603 binds directly to the MGMT 3'UTR and results in a loss of MGMT protein expression both *in vitro* and *in vivo*. In addition, we show that miR-603 and miR-181d cooperate to regulate MGMT expression.

## RESULTS

### Identifying miRNAs that suppress MGMT expression

**Fig1:**
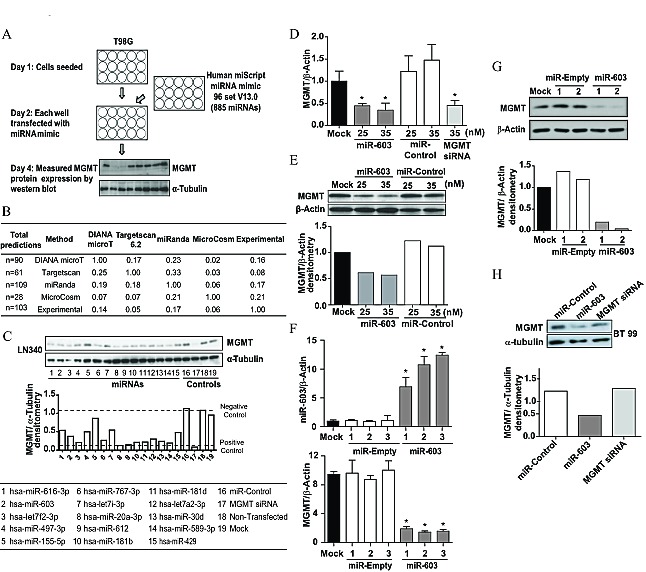
MGMT expression is silenced by miR-603 (A) Schema of screen to identify miRNAs that suppressed MGMT expression. A library of 885 miRNAs were transfected into T98G cells 24 hours after seeding (cells were seeded at 1,000 cell/well). Lysates were prepared 48 hours after transfection and MGMT expression was quantified by Western blotting [43]. (B) Comparing screen results with predictions of computational algorithms. Predicted miRNAs that bind to the 3'UTR of MGMT were identified using four different algorithms (miRanda, DIANA microTv3.0, Targetscan 6.2 and MicroCosm). Results were compared in a pairwise manner between algorithmic predictions and our screen results. The set of miRNAs that overlapped between methods are shown as a fraction of the total number of predicted miRNA shown to the left (under the column Total predictions).The overlap was greatest between MicroCosm and the screen result. (C) Identification of potential MGMT regulating miRNAs. miRNAs that suppressed MGMT expression in the screen were tested to see whether their expression inversely correlated with MGMT expression in MGMT promoter unmethylated glioblastomas using the Chinese Glioma Genome Atlas (CGGA). Candidates that exhibited inverse correlation commensurate with miR-181d, an established MGMT-regulating miRNA [17] were selected for further characterization. These 15 miRNAs were transfected into LN340 glioblastoma cells. 48 hours after transfection, MGMT silencing was assessed by Western blotting; α-tubulin was used as a loading control. Quantitative assessments of MGMT expression are shown in the bottom panel. (D) qRT-PCR demonstrating MGMT silencing 72 hours after transfection of A1207 cells with miR-603 mimic. No change was observed with non-targeting control miRNA; MGMT siRNA was used as positive control. (E) Western blot confirmation of MGMT silencing by miR-603 mimic transfection in A1207cells. Lysates were prepared 72 hours after transfection. Quantitative assessments of MGMT expression are shown in the bottom panel. (F) Stable miR-603 expression suppressed MGMT expression. A miR-603 expressing construct (pCMV-miR-603) or vector control was transfected into LN340 and passaged in Neomycin for selection of stable integrants. Independent clones from these transfections were tested for miR-603 and MGMT expression by qRT-PCR. (G) Western blotting confirmed that miR-603 expression suppressed MGMT expression. Independent clones of miR-603 and vector transfected LN340 were analyzed for MGMT expression. The clones tested here are the same as those tested in 1F. Quantitative assessment of MGMT expression is shown in the right panel. (H) miR-603 transfection of the BT99 neurosphere line caused suppression of MGMT expression. MGMT siRNA was used as a positive control. Quantitative assessment of MGMT expression is shown in the right panel.

To identify miRNAs that suppressed MGMT expression, we individually transfected 885 known miRNAs (Human miScript miRNA mimic 96 set, Qiagen) into T98G, a glioblastoma cell line that showed high expression of MGMT. 48 hours after transfection, the cells were assessed for viability by direct visual inspection (Supplementary Figure 1). MGMT protein expression was then measured by Western blotting (Figure 1A). miRNAs that suppressed MGMT expression more than 50% without significant cytotoxicity (>50% cell death) were identified. A total of 103 miRNAs were identified in this manner (Supplementary Table 1).

We first tested whether the identified miRNAs coincided with those predicted to interact with the 3'UTR of MGMT by differing algorithms. For this *in silico* analysis, only miRNAs predicted with a total context score of -0.25 or lower were considered. The number of miRNAs predicted to bind to MGMT-3'UTR by DIANA microTv3.0 [18], Targetscan 6.2 [19], miRanda [20], and MicroCosm [21] were 90, 61, 109, and 28 respectively (Figure 1B). Overall, there was very little concordance (3-22%) between the predictive algorithms and our experimental results. Of the four algorithms tested, MicroCosm predicted the greatest number of miRNAs uncovered in our screen.

### miR-603 as an MGMT-regulating miRNA

From the 103 miRNAs that suppressed MGMT expression *in vitro*, we wanted to identify those that regulate MGMT expression in glioblastomas. We reasoned that these miRNAs should exhibit an inverse correlation with MGMT expression level in MGMT promoter unmethylated specimens [22]. In order to identify these miRNA, we used the Chinese Glioma Genome Atlas (CGGA) database. We identified miRNAs with inverse correlation coefficients comparable to miR-181d, a previously identified MGMT-regulating miRNA (See Methods). The 15 candidates that fulfilled this criterion were further tested for their effects on MGMT expression in LN340, an MGMT expressing glioblastoma cell line (Figure 1C, Supplementary Figure 2).

Previous reports suggest miR-221/mir-222 [23], miR-767-3p, and miR-648 [24] as potential MGMT regulating miRNAs in glioblastoma. However, miR-221, miR-222 and miR-648 transfection did not consistently suppressed MGMT expression in our screen. Moreover, their expression did not inversely correlate with MGMT expression (Supplemental Figure 3). In contrast, miR-767-3p transfection suppressed MGMT expression (Figure 1C). Its expression level also inversely correlated with MGMT mRNA expression.

Of the top 15 candidates identified by our screen, miR-603 most strongly suppressed MGMT expression in all cell lines tested. When miR-603 mimics were transfected into A1207 cells, MGMT mRNA and protein were suppressed by approximately 50% (Figure 1D, E). To further confirm the MGMT suppressing effect of miR-603, we established an LN340 cell line that stably expressed miR-603 (see Methods). Independent clones of miR-603 and empty vector containing lines were tested by quantitative real time PCR (qRT-PCR) for MGMT expression. In this stable expression model, miR-603 expression was consistently associated with approximately 6-fold decrease in MGMT mRNA and protein levels (Figure 1F, G).

Emerging literature suggests that neurosphere culturing conditions better simulate glioblastoma biology than adherent lines [25]. Therefore, we used neurosphere lines as a model system to confirm the suppression of MGMT by miR-603. In BT99 neurosphere cells, transfection of miR-603 mimics caused an approximately 3-fold decrease in MGMT protein expression (Figure 1 H). Together, these data suggest that miR-603 suppressed MGMT expression.

### Physiologic regulation of MGMT by miR-603

**Fig 2:**
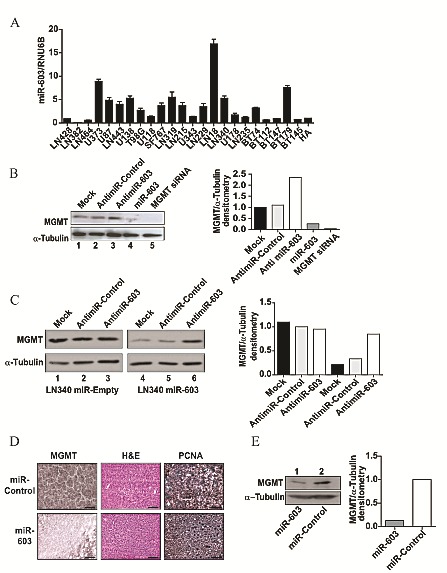
miR-603 regulation of MGMT is specific and reversible (A) Expression levels of miR-603 in 23 glioblastoma lines (19 passaged as adherent culture in serum and 5 passaged as neurospheres). LN18 cells showed high basal expression of miR-603. (B) Anti-miR-603 transfection increased MGMT expression in the LN18 glioblastoma line. MGMT protein expression was assessed 48 hours after anti-miR-603 transfection. (C) Anti-miR-603 transfection increased MGMT expression in the LN340 glioblastoma lines over-expressing miR-603. miR603-expression and vector transfected clones described in Figure 1F were transfected with anti-miR-603 or non-targeting miRNA control. MGMT expression was assessed 48 hours after transfection. Quantitative assessment of MGMT expression is shown in the right panel. (D) Intra-tumoral injection of miR-603 mimic suppressed MGMT expression in vivo. Flank murine GBM43TMZ xenograft tumors (4 tumors per condition) [26] were injected with two doses of miR-603 24 hours apart after the xenograft reached 50mm^3^ in size. Xenografts were harvested 48 hours after injection and processed for H&E staining, PCNA immuno-staining (as positive control [51]), and MGMT immuno-staining. Three independent experiments were performed on independent days. Representative data is shown. Scale bar is 50μm. (E) Western blotting confirmed that intra-tumoral injection of miR-603 suppressed MGMT expression in vivo. Samples described in Figure 2D were processed for MGMT Western blotting. Quantitative assessment of MGMT expression is shown on the right panel.

A major concern with transfection or exogenous expression of miRNA involves the possibility that these manipulations result in non-physiologically high concentrations of the miRNA, resulting in experimental artifacts. In order to exclude this possibility, we identified a glioblastoma line, LN18, which intrinsically expressed miR-603 at a level 18-fold higher than other cell lines tested (Figure 2A). We tested whether the neutralization of miR-603 via antimiR transfection in this cell line increased MGMT silencing. When LN18 cells were transfected with anti-miR-603, a 2.3-fold increase of MGMT protein expression was observed (Figure 2B). This effect was not observed when cells were transfected with a control anti-miR. We recapitulated this result using LN340 lines stably expressing miR-603. Transfection of these lines with anti-miR-603 resulted in an approximately 4-fold increase in MGMT protein expression. This effect was not observed with a control anti-miR or in LN340 lines transfected with a vector control (Figure 2C). These results suggest that physiologic levels of miR-603 modulate MGMT expression.

We next determined whether miR-603 suppressed MGMT expression *in vivo*. High MGMT expressing, patient-derived glioblastoma xenografts (GBM12TMZ and 43TMZ) that were serially passaged in murine flank were injected with miR-603 or non-targeting miRNA (4 tumors per condition) [26]. 48 hours after injection, xenografts were harvested, and MGMT expression was assessed by IHC and Western blotting. GBM12TMZ injected with miR-603 exhibited no detectable MGMT staining by IHC, whereas intense MGMT staining was found in xenografts injected with the control miRNA (Figure 2D). Western blotting analysis showed that the miR-603 injected xenograft exhibited an approximately 8-fold decrease in MGMT expression relative to the control injected xenograft (Figure 2E). Similar results were observed with GBM43TMZ, suggesting that the introduction of miR-603 *in vivo* suppressed MGMT expression.

### Interaction between miR-603 and the MGMT 3'UTR

**Fig 3:**
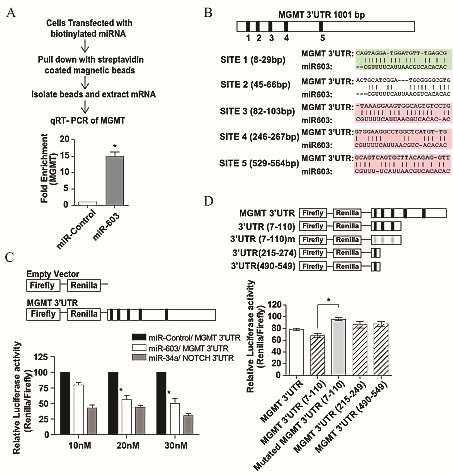
miR-603 directly interacts with the 3'UTR of MGMT (A) MGMT mRNA co-precipitated with biotinylated miR-603. 48 hours after biotinylated miR-603 or biotinylated non-targeting miRNA (30nM) was transfected into A1207 cells, cells were lysed and treated with streptavidin coated magnetic beads. qRT-PCR was performed to determine the relative abundance of MGMT mRNA and GAPDH mRNA (control). There was a significant enrichment of MGMT mRNA in the biotinylated miR-603 pull-down relative to the non-targeting miRNA pull-down. (B) Predicted miR-603 binding sites (MREs) in 3'UTR of MGMT. MRE prediction was performed using Targetscan 4.2. (C) Co-transfection of a luciferase reporter vector with the full length MGMT 3'UTR and miR-603 mimics resulted in a significant loss of luciferase activity. A1207 cells were seeded at a 5x10^5^ cells per well. 24 hours after seeding, cells were transfected with both miR-603 and the MGMT 3'UTR or non-targeting miRNA and the MGMT 3'UTR. The NOTCH 3'UTR was co-transfected with miR-34a as a positive control. Luciferase activity was assessed 48 hours after co-transfection. (D) Mutation of miR-603 MRE in the MGMT 3'UTR abolished the suppressive effect of miR-603. Truncated versions of the MGMT 3'UTR were constructed and tested as above described. Mutations of the first three MREs disrupted the luciferase suppressive effect of miR-603.

We wished to determine whether miR-603 suppresses MGMT by direct interaction with the MGMT 3' UTR [27]. For this purpose, 3'biotinylated miR-603 or non-targeting miRNA were transfected into A1207 glioblastoma cells as described previously [17]. mRNA-biotinylated miRNA complexes were pulled down using streptavidin coated magnetic beads and bound MGMT and GADPH mRNA were analyzed by quantitative RT-PCR (Figure 3A). We detected a 15-fold enrichment of MGMT in the biotinylated miR-603 pull-down relative to biotinylated non-targeting miRNA. A GAPDH mRNA pull-down using a GAPDH miRNA was performed as a positive control. The mRNAs that were pulled-down by GAPDH miRNA were not enriched for MGMT. We also did not detect any GAPDH mRNA in the miR-603 pull-down (data not shown).

Additionally, we identified 5 potential MREs (miRNA Response Elements) for miR-603 in the MGMT 3'UTR (Figure 3B). The luciferase activity of a pSiCheck-2 reporter construct harboring the MGMT 3'UTR was reduced by 40% when co-transfected with miR-603 relative to experiments where co-transfection was performed using non-targeting miRNAs (Figure 3C). Moreover, this suppressive effect was abolished when the first three putative miR-603 MREs were mutated (Figure 3D). These results suggest that miR-603 modulates MGMT expression via interaction with the MGMT 3'UTR.

### miR-603 sensitizes cells to temozolomide treatment

**Fig4:**
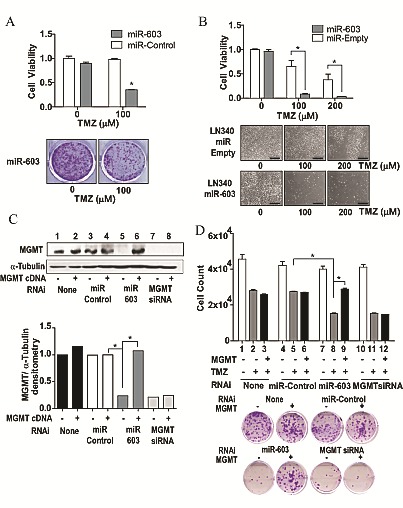
miR-603 sensitizes GBM cells to treatment with TMZ (A) Transient transfection with miR-603 mimics sensitizes A1207 cells to TMZ treatment. Cells were seeded at 1,000 cells per well. 24 hours after seeding, cells were transfected with 30nM miR-603 or non-targeting miRNA. 24 hours after transfection, the cells were washed with PBS and TMZ containing media was added. Clonogenic survival was assessed 14 days after transfection. Representative images of the survival assay are shown in the lower panel. (B) Stable expression of miR-603sensitizes LN340 to TMZ treatment. LN340 with stably integrated pCMV-miR-603 or vector control (described in Fig 1F) were tested for TMZ sensitivity. Representative images of the assay are shown in the lower panel. Scale bar is 50μm. (C) The MGMT-suppressive effect of miR-603 was rescued by MGMT cDNA expression. A1207 cells were seeded at 5×10^5^ cells per well. 24 hours after seeding, cells were co-transfected with combinations of miR-603 mimic (60nM) + MGMT cDNA or miR-603 mimic (60nM) + empty vector. Cells were lysed 48 hours after transfection and assessed for MGMT expression. α-tubulin was used as a control. Quantitative assessment of MGMT expression was shown in the lower panel. (D) The TMZ sensitizing effect of miR-603 was rescued by MGMT cDNA expression. The experiments were performed in parallel to those described in Figure 4C. After the various co-transfections, cells were seeded with or without TMZ treatment. Clonogenic survival was determined 14 days after TMZ treatment. Representative images of the clonogenic survival assays are shown in the lower panel.

Modulation of MGMT expression should translate into altered temozolomide sensitivity [28]. We first tested this prediction using transient transfection assays. A1207 cells were transfected with miR-603 or non-targeting miRNA and then treated with temozolomide. Cells transfected with miR-603 showed a 60% loss in temozolomide resistance (Figure 4A) relative to cells transfected with non-targeting miRNA. Additionally, LN340 cells stably expressing miR-603 demonstrated a near two order of magnitude increase in temozolomide sensitivity relative to LN340 stably transfected with a vector control (Figure 4B). Importantly, co-transfection of MGMT cDNA (missing the 3'UTR of MGMT) with miR-603 reversed the expression and temozolomide sensitization effects of miR-603 on MGMT (Figure 4C lane 5 versus 6 and Figure 4D). Expectedly, the MGMT cDNA was not able to reverse the effect of an siRNA targeting the coding sequence of MGMT (Figure 4C, lanes 7-8).

### miR-603 cooperates with miR-181d to silence MGMT

**Fig 5:**
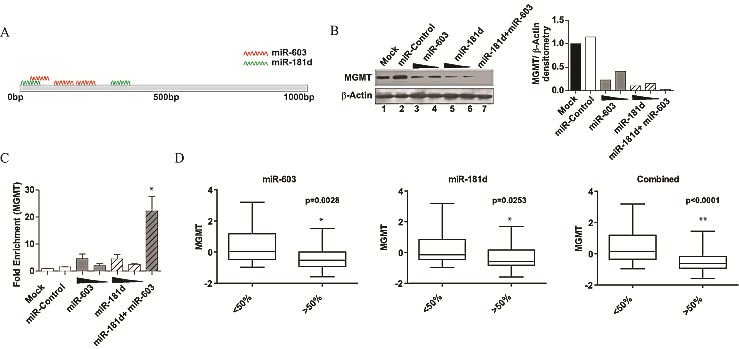
miR-603 and miR-181d act cooperatively to silence MGMT (A) Schematic representation of the predicted miR-181d and miR-603 binding sites on MGMT 3'UTR. MRE prediction was performed by Targetscan 4.2. The miR-181d MREs were previously published [17]. The first three miR-603 MREs are as shown in Figure 3D. (B) miR-603 and miR-181d cooperate to suppress MGMT expression. A1207 cells were seeded at 5x105 cells per well. 24 hours after seeding, cells were transfected with miR-603 (25nM or 30 nM), miR-181d (5nM, 30nM) or a combination of miR-603(25nM) and miR-181d (5nM). Non-targeting miRNA (30nM) was transfected as a control. MGMT expression was assessed by Western blotting 72 hours after transfection. Quantitative assessment of MGMT expression is shown on the right panel. (C) Cooperative binding of miR-603 and miR-181d to MGMT mRNA. Biotinylated miR-603 (15nM or 20nM) or biotinylated miR-181d (5nM, 20nM) were transfected in A1207 cells. Biotinylated non-targeting miRNA (20nM) were transfected as a control. 48 hours after transfection, cells were lysed and treated with streptavidin coated magnetic beads. An increase in MGMT mRNA precipitation is seen with a combination of biotinylated miR-603 and biotinylated miR-181d. This effect is not observed with increasing concentrations of either miRNA by itself. (D) An index of miR-181d and miR-603 more closely mirrors MGMT expression in glioblastoma specimens than each individual miRNA. To avoid arbitrary cut offs, we examined MGMT expression in the specimens dichotomized by the median expression of miR-181d, miR-603, or a combined index of miR-181d and miR-603. Student's t-test was performed to assess whether MGMT expression differed significantly between the miR-high and miR-low groups. P-values of the comparison are as shown.

Cooperative repressive effects have been documented between different miRNAs with distinct MRE sites on a target gene [29-32]. We wished to determine if miR-181d and miR-603 (Figure 5A) exhibit such an interaction [33, 34]. We titrated the amount of miR-181d and miR-603 necessary to achieve the same level of MGMT repression (lanes 4 and 6, Figure 5B). The transfection of 5nM miR-181d suppressed MGMT expression by 90%, while 25nM of miR-603 suppressed MGMT by 78%. Co-transfection of LN340 with 25nM miR-603 and 5nM miR-181d completely suppressed MGMT expression. Of note, this complete suppression was not achieved by 30nM of miR603 or 30nM of miR-181d, suggesting cooperativity between these two miRNAs in their interactions with MGMT (lanes 3, 5 versus 7).

We next tested whether this cooperativity occurred at the level of mRNA interaction. To test this hypothesis, we performed affinity pull-down experiments with a combination of biotin tagged miR-181d and miR-603. At 5nM concentration, biotinylated-miR-181d pull-down showed a 2.4-fold enrichment in the level of MGMT mRNA relative to a control-miR pull-down (Figure 5B). A similar level of MGMT-mRNA enrichment was observed with the miR-603 pull-down relative to the control miR pull-down. Notably, when a mix of 5nM biotinylated-miR-181d and 15nM biotinylated-miR-603 were used, the pull-down showed a 22-fold enrichment in MGMT mRNA relative to the control miR pull-down (Figure 5D). This synergistic enrichment was not observed when pull-downs were performed with 20nM of biotinylated-miR-181d or 20nM of biotinylated-miR-603. These results support a model of cooperative binding between miR-603 and miR-181d to the MGMT mRNA.

These results suggest that MGMT expression is regulated by multiple miRNAs. This model predicts that a combined index of miR-181d and miR-603 should better predict MGMT expression levels than either miRNA individually. MGMT, miR-181d and miR-603 levels were characterized for 74 glioblastoma specimens from the UCSD glioblastoma tumor bank by qRT-PCR; GAPDH was characterized as a control. We divided the specimens into 2 groups based on median miR-181d or miR-603 expression levels. For both miR-603 and miR-181d, the difference in the MGMT expression levels between the high versus low groups was statistically significant (p=0.0028 and p=0.0253 respectively) (Figure 5D). A combined index of miR-181d and miR-603 expression levels captured the variation in MGMT expression better than each individually. For the index, the difference in MGMT expression between the high and low miRNA samples was highly significant at p<0.0001. In aggregate, the co-transfection studies, the biotin pull-down studies, and the clinical specimen analysis support a model where MGMT expression is regulated by miR-603 and miR-181d.

## DISCUSSION

Here, we present the first study to integrate a genome-wide miRNA screen with an informatics screen using genomic data derived from clinical specimens to identify clinically pertinent MGMT-regulating miRNAs. We previously demonstrated that MGMT is post-transcriptionally regulated by miRNAs, specifically miR-181d [16, 17]. Through a screen that utilized MGMT expression from endogenous loci as an assay, we screened for other miRNAs that suppressed MGMT expression. To our surprise, nearly a fifth of transfected miRNAs directly or indirectly modulated MGMT protein expression. We then hybridized this dataset to identify the miRNAs whose expression inversely correlated with the expression of MGMT in MGMT promoter unmethylated glioblastomas. This refined our list of miRNA candidates to fifteen. This result suggests that most of the MGMT-regulating miRNA identified in a cell-based assay are unlikely to be pertinent and emphasize the need to integrate the results of such screens with clinical genomic information. We characterized one candidate in detail, miR-603, and showed that it binds to the MGMT 3'UTR to down-regulate MGMT. Importantly, miR-603 cooperated with miR-181d to regulate MGMT expression. In clinical specimens, a combined index of the two miRNA better reflects MGMT expression than the expression of either miRNA individually

Our near-exhaustive screen of MGMT regulating miRNAs affords an opportunity to compare experimental data with *in silico* prediction algorithms. While there are several computational prediction algorithms available to predict miRNA–mRNA interactions [18-21], the degree of overlap of their individual predictions is poor [35, 36]. The discrepant predictions between algorithms were again seen when we analyzed the 3'UTR of MGMT. The predictions that were most consistent with our results were generated by MicroCosm, where 21% of the miRNAs that were predicted to interact with MGMT 3'UTR also suppressed MGMT expression. It is notable that many of the miRNAs that suppress MGMT expression were not predicted to bind to MGMT 3'UTR by any of the algorithms. Since we had excluded miRNAs with significant cytotoxicity from our analysis, this result suggests that a large number of miRNAs can influence expression of a gene without direct binding to the mRNA of that gene. For instance, MGMT degradation is mediated through proteasomes [37] and miRNAs that modulate proteasome function may “indirectly” influence MGMT levels. As such, the miRNA regulatory network for MGMT regulation may be significantly more complex than models with focus on miRNAs that bind to the target gene. While these results were derived using MGMT as a model, the finding may bear relevance to miRNA regulation of oncogenes or tumor suppressors genes.

In addition to the complexity of indirect miRNA regulators, our results further suggest that MGMT is concordantly regulated by at least two miRNAs that bind to its 3'UTR, miR-181d and miR-603. In general, stronger miRNA- mediated repression of mRNA is observed when there are multiple binding sites for the same miRNA on a 3'UTR [29, 33]. Moreover, several recent reports have demonstrated cooperative binding of different miRNAs on the same 3'UTR [30, 34, 38]. This cooperativity requires that the involved miRNA response elements (MREs) be placed 16-40 nucleotides apart in the 3'UTR [32, 34]. Consistent with these findings, three of the five binding sites predicted for miR-603 on the MGMT 3'UTR are located within 40bp of miR-181d MREs. The arrangement of these MREs likely contributed to regulation of MGMT by miR-603 and miR-181d.

Only approximately 10% of glioblastoma patients derive durable benefit from temozolomide treatment [39, 40]. However, temozolomide remains a standard of care for all glioblastoma patients because there are currently no reliable predictive biomarkers of response [41]. As temozolomide is a mutagen, it is highly desirable that patients who will not derive durable benefit be excluded from this therapy [42]. While MGMT promoter methylation status represents a significant advance in this regard, a significant portion of MGMT promoter unmethylated patients derive therapeutic benefit from temozolomide. A contributing factor to the limitation of MGMT promoter assays is the post-transcriptional regulation of MGMT. For instance, an MGMT promoter unmethylated glioblastoma may harbor low MGMT expression as a result of high miR-181d or miR-603 expression. Therefore, an integrated platform which incorporates MGMT promoter methylation with MGMT-regulating miRNA expression may be able to better predict temozolomide therapeutic response.

## MATERIALS AND METHODS

### Cell culture and stable cell lines

Human glioblastoma cell lines A1207, LN340, LN18, LN428, LN382, LN464, U373, U87, LN443, U138, U118, SF767, LN319, LN215, U343, LN229, U178, LN235 and T98G were cultured in DMEM containing 10% FBS and 1% Penicillin-Streptomycin (Invitrogen, Carlsbad, CA) at 37°C in a 5% CO_2_ incubator and passaged with Trypsin. NHA were purchased from ScienCell (Carlsbad, CA). NHA were passaged using Astrocyte Medium from ScienCell. The BT99, BT74, BT112, BT147, BT179, BT145 neurosphere line was provided by Dr. Keith Ligon (Dana Farber Cancer Institute). Neurosphere lines were cultured in NeuroCult media (StemCell Technologies, Vancouver, BC, Canada) supplemented with EGF, FGF and Heparin per manufacturer's instructions and grown at 37°C in a 5% CO_2_ incubator. Stable cell lines were established by transfecting LN340 cells with the pCMV-miR-603 miRNA expression vector or empty vector control (SC400580, Origene, Rockville,MD). Stable cells were selected using neomycin. Single clones were isolated and cultured in DMEM containing 10% FBS and 1% Penicillin-Streptomycin (Invitrogen) at 37°C in a 5% CO_2_ incubator.

### miRNA screen

T98G cells were seeded into 96 well plates at 1,000 cells per well in 80μl of medium. 24 hours after seeding, cells were transfected with 885 Human miScript miRNA mimics (Qiagen, Valencia, CA) in 96 well plates. Transfection was performed by the addition of a transfection mix consisting of 15.5μl of Opti-MEM, 0.5μl of Hiperfect (Qiagen), and 4μl of 2μM siRNA. 48 hours after transfection, miRNA cytotoxicity was scored by visual inspection under the microscope, Qiagen cell death siRNA used as a positive control for comparison on each 96 well plate. miRNAs causing >50% reduction in cell number were excluded from further consideration. Cell extracts were prepared from the 96 plates as described previously [43].

### Western blotting

Cells were lysed in RIPA buffer containing 20mM Tris–HCl (pH 7.4), 150mM NaCl, 1mM EDTA, 1% Triton-X 100, 20mM β-glycerophosphate and 1mM p-amidinophenyl methanesulfonyl fluoride hydrochloride supplemented with complete Protease Inhibitor Cocktail (Roche, Madison, WI). The lysates were heated at 95°C for 5 minutes and cleared by centrifugation at 12,000rpm for 10 minutes before immunoblotting analysis. Immunoblotting was performed using MGMT antibody (Cell Signaling Technology, ab7045, 1:500). β-actin (Thermo Scientific, Lafayette, CO, 01673088,1:4000) and α-tubulin (Sigma, T9026, 1:4000) were used as loading controls. Intensities of the protein bands were quantified using ImageJ software [44].

### Transfection of miRNA mimics and antimiRs

Cells were transfected using HiPerfect transfection reagent (Qiagen) with miRNA mimics (miR-603: MSY0003271, miR-181d: MSY0002821, Allstars Negative control siRNA: 1027280 (Qiagen), MGMT SMARTpool siRNA: M-008856-00-0005 (Dharmacon, Pittsburgh, PA)) or antimiRs (Anti-miR-603:MIN0003271, anti-miR-181d: MIN0002821, anti-miR-Control 1027271) from Qiagen per manufacturer's protocol. Total RNA and protein were extracted from cells after 72 hours and analyzed by Western blotting or qRT-PCR.

### Real-time PCR analysis

Total RNA was extracted from cells using miRNeasy kit (Qiagen) according to the manufacturer's protocol. cDNA was synthesized using 0.5 μg of total RNA with the iScript cDNA synthesis kit (Bio-Rad, Hercules, CA) or Exiqon cDNA synthesis kit (Exiqon, Woburn, MA). miRNA and mRNA transcripts were quantified using SYBR Green (Bio-Rad) on the Bio-Rad Chromo 4 DNA Engine Thermal Cycler. The primers used are listed below:

hsa-miR-603: Catalog number 204112, Exiqon (Woburn, MA); Catalog number 001566, Life technologies (Grand Island, NY)

hsa-miR-181d: Catalog number 204789, Exiqon (Woburn, MA)

RNU6B: Catalog number 001093, Life technologies (Grand Island, NY),

MGMT Forward: 5' CCTGGCTGAATGCCTATTTC 3'

MGMT Reverse: 5' GATGAGGATGGGGACAGGATT 3'

Actin Forward: 5' TGAAGTGTGACGTGGACATC 3'

Actin Reverse: 5' GGAGGAAGCAATGATCTTGAT 3'

GAPDH Forward: 5'ATCATCCCTGCCTCTACTGG 3'

GAPDH Reverse: 5'GTCAGGTCCACCACTGACAC 3'

### Glioblastoma xenografts

All animal studies were performed in accordance with the Animal Care and Use Rules at the University of California San Diego. Primary glioblastoma xenograft lines with a “GBM” prefix were kindly provided by Dr. Jann Sarkaria at the Mayo Clinic. These tumor samples were originally derived from patient surgical specimens and serially passaged as subcutaneous xenograft tumors [45]. GBM12TMZ and GBM43TMZ were derived from GBM12 and GBM43 after *in vivo* treatment with TMZ. In these two xenografts, a significant induction of MGMT expression was observed after TMZ treatment [26]. GBM xenograft tumors were established in the flank of 5 week old nude mice as described previously [26]. Once solid tumors reached 50mm^3^ in size, 25μl of miR-603 mimics or non-targeting control miRNA were injected into the tumor (4 tumors each). Each injection contained 250pmol of mimics, and mice received two injections of miR-603 or non-targeting control miRNA 24 hours apart. 48 hours after the final injection, mice were sacrificed and tumors were prepared for histology or protein isolation.

### Immunohistochemical staining

Immunohistochemistry (IHC) staining was carried out on 4μm sections heated for 30 minutes at 60°C using the Bond III fully automated staining system with their Bond Polymer Refine detection system and associated reagents supplied by Leica Microsystems (Newcastle-Upon-Tyne, UK). Antigen retrieval and dilution was performed at 100°C for 30 min with MGMT antibody (Millipore, Thermo Fisher, UK) or PCNA antibody (Cell Signaling Technologies, 2586s) at a 1:100 dilution with Epitope Retrieval Solution 1(pH 6.0). Primary antibodies were applied to the section for 30 minutes. Slides were deparaffinized and pretreated with steam disodium ethylene diamine-tetraacetate at pH 8.0. A 30 minute incubation time with the primary antibody was performed. The primary antibody was substituted with mouse IgG1 at a dilution of 1:200 as a negative control. In accordance with previous reports, MGMT was considered positive when uniform MGMT staining was detected in cell nuclei [46].

### miRNA affinity tagged precipitation

40nM of 3' biotinylated miR-603 or control miRNA (cel-miR-67) (Thermo Scientific) was transfected into A1207 glioblastoma cells using Lipofectamine RNAiMax (Invitrogen) as described previously [17]. GAPDH miRNA was also transfected as a positive control to precipitate GAPDH mRNA. 48 hours after transfection, cells were lysed using Lysis Buffer: 700μl of 20mM Tris (pH7.5), 100 mM KCl, 5 mM MgCl2, 0.3% Nonidet P-40, 50U RNAseOUT (Invitrogen) and 1:50,000 Protease Inhibitor Cocktail (Roche). After centrifugation (10,000xg, 10 min), the supernatant was mixed with streptavidin-coated magnetic beads (Invitrogen, Carlsbad, CA) for 4 hours at 4°C. The beads were washed, and the bound mRNA was extracted using the miRNAeasy kit (Qiagen) as pull-down. cDNA was synthesized from the pull-down using MMLV-RT (Epicenter Biotechnology, Madison, WI) and random primers (Promega, Madison, WI). qRT-PCR was then performed and the fold enrichment of MGMT transcript in the pull-down was determined for both control miRNA and miR-603.

### Clonogenic assay

Cells were plated at a concentration of 500 cells per well. The following day, cells were treated with temozolomide or DMSO as a control. 14 days after treatment, cells were rinsed with PBS, fixed with acetic acid: methanol (1:3) for 15 minutes and stained with crystal violet; and the number of colonies formed per well were counted. A colony is defined as having 50 or more cells.

### Reporter Assays and Cloning

The MGMT 3'UTR (1,001 bp) was amplified from genomic DNA isolated from U87 glioblasoma cells and cloned into pSiCheck-2 dual reporter vector (Promega). Primers used are shown below. A1207 glioblastoma cells were co-transfected with miR-603 or non-targeting miRNA with either pSi-Check-2-MGMT 3'UTR or empty vector controls using Lipofectamine 2000 (Invitrogen) per manufacturer's instructions. The MGMT 3'UTR was sectioned into three regions (7110bp, 215-274bp and 490-549bp) containing predicted miRNA binding sites based on restriction site availability. These three regions were cloned into pSiCheck-2 luciferase reporter vector. Corresponding constructs with mutated MREs were also cloned. All cloned sequences were verified by direct DNA sequencing.

PCR primers used to amplify the 1,001 bp of MGMT 3'UTR:

MGMT 3'UTR FP: ACTCGAGTGCAGTAGGATGGATGTTTGA

MGMT 3'UTR RP: AGCGGCCGCATGCAGAGCTACAGGTTTCC

### Temozolomide sensitivity assays and rescue experiments

Cells were seeded at a concentration of 1000 cells per well. 24 hours after seeding, cells were transfected with 30nM miR-603 or non-targeting miRNA. 24 hours after transfection, the cells were washed with PBS and TMZ containing media was added. Clonogenic survival was assessed 14 days after transfection.

A1207 cells were seeded at 5x10^5^ cells per well. 24 hours after seeding, cells were co-transfected with combinations of miR-603 mimic (60nM) + MGMT cDNA or miR-603 mimic (60nM) + empty vector. The MGMT cDNA vector was lacking the 3'UTR. Cells were lysed 48 hours after transfection and assessed for MGMT expression

### Glioblastoma patient specimen and analysis

All research performed was approved by IRB boards at University of California, San Diego Human Research Protections Program and was in accordance with the principles expressed at the declaration at Helsinki. Each patient was consented by a dedicated clinical research specialist prior to collection. Written consent was obtained for each patient. The consent process was approved by the ethics committee, and all records were documented in our electronic record system. The written consent from patients was also scanned into our electronic record system. All diagnoses of glioblastoma were histologically confirmed. Glioblasotma specimens were further characterized in terms of qRT-PCR for the expression levels of miR-181d, miR-603, and MGMT. 18S rRNA, GAPDH and Actin were used as reference genes, yielding comparable results.

### Bioinformatic and statistical analyses

In order to computationally determine all the miRNAs that target MGMT, we used four of the leading miRNA prediction algorithms: DIANA - microT v3.0 http://diana.cslab.ece.ntua.gr/microT/ [18, 47], miRanda http://www.microrna.org/microrna/getGeneForm.do [20, 48], MicroCosm http://www.ebi.ac.uk/enright-srv/microcosm/htdocs/targets/v5/ and Targetscan 6.2 http://www.targetscan.org/ [19, 49]. TargetScan 4.2 was used to identify predicted binding sites for miR-603 on the MGMT 3'UTR.

The CGGA data set was kindly provided by Dr. Tao Jiang (Department of Neurosurgery, Tiantan Medical Center, Beijing, China) as normalized, probe-level expression values. Values of probes designed to assess the same gene were averaged. MGMT promoter methylation status was determined as previously described [50]. The expression levels of MGMT mRNA and candidate miRNAs were analyzed by Pearson's correlation. Candidate miRNAs that negatively correlated with MGMT mRNA levels with Pearson's correlations coefficients greater than or equal to that observed for miR-181d (R^2^= −0.2) were selected for further analysis.

MGMT mRNA expression, MGMT methylation, and miRNA expression were downloaded from the TCGA Data Portal (http://tcga-data.nci.nih.gov/tcga/) as level 3, normalized data. Only patients with complete information were included for the analysis (n = 214). MGMT methylation status (probe cg12981137) was binarized using the mean value as a cut –off. Patient with a score lower than the mean were considered as unmethylated. The expression levels of MGMT mRNA and candidate miRNAs were analyzed by Pearson's correlation.

Statistical analyses were performed using the GraphPad Prism Software version 5 (GraphPad, La Jolla, CA). Data are presented as the means with their respective standard errors (SEM). Student's *t* test (two-tailed) was used and p < 0.05 was considered to be statistically significant.

## SUPPLEMENTARY FIGURES AND TABLES


