# Supplementary material for: A genome-wide miRNA screen revealed miR-603 as a MGMT-regulating miRNA in glioblastomas
Source: Oncotarget. 2014 May 14;5(12):4026–39. doi: 10.18632/oncotarget.1974 (PMC4147303; doi:10.18632/oncotarget.1974)
Supplement: Supplementary file 1 [file oncotarget-05-4026-s001.pdf]

A genome-wide miRNA screen revealed miR-603 as a MGMT-regulating miRNA in glioblastomas

Supplementary Material

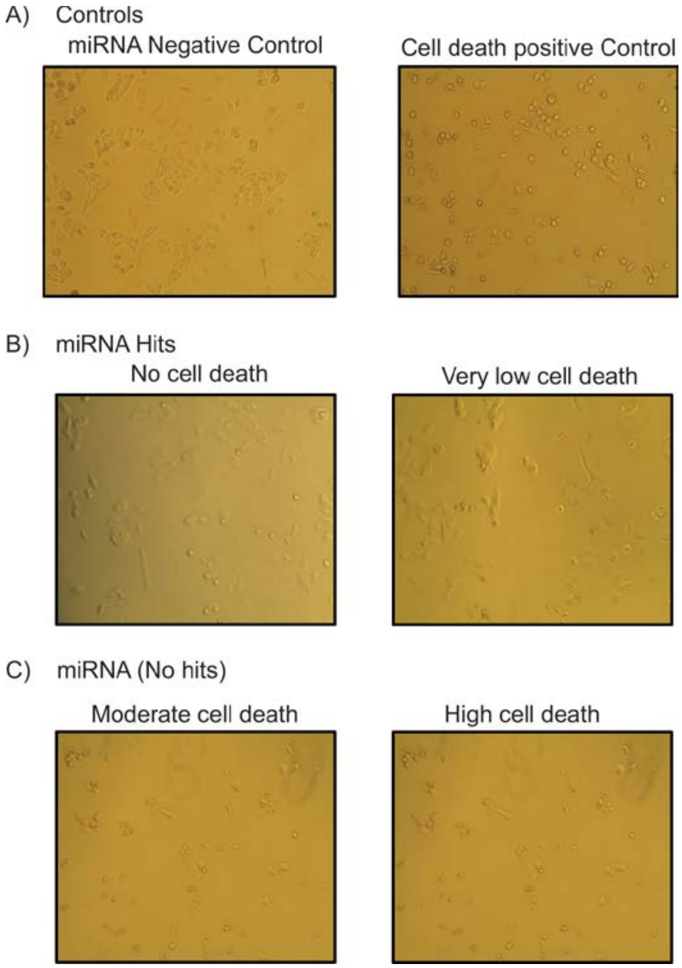

Supplementary Figure 1: Representative images depicting miRNA toxicity from the screen. Toxicity was scored by visual inspection, Qiagen cell death siRNA was used as a positive control for comparison on each 96 well plate.

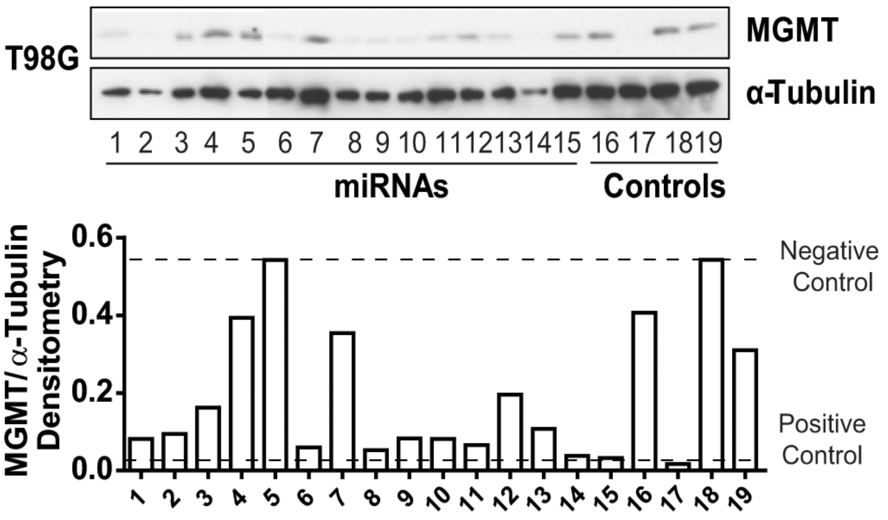

1 hsa-miR-616-5p	6 hsa-miR-767-3p	11 hsa-miR-181d	16 miR-Control
2 hsa-miR-603	7 hsa-let7i-3p	12 hsa-let7a2-3p	17 MGMT siRNA
3 hsa-let7f2-3p	8 hsa-miR-20a-3p	13 hsa-miR-30d	18 Non-Transfected
4 hsa-miR-497-3p	9 hsa-miR-612	14 hsa-miR-589-3p	19 Mock
5 hsa-miR-155-5p	10 hsa-miR-181b	15 hsa-miR-429	

Supplementary Figure 2: 15 candidate miRNAs were transfected into T98G glioblastoma cells. 48 hours after transfection, MGMT silencing was assessed by Western blotting; α-tubulin was used as a loading control. Quantitative assessments of MGMT expression are shown in the bottom panel.

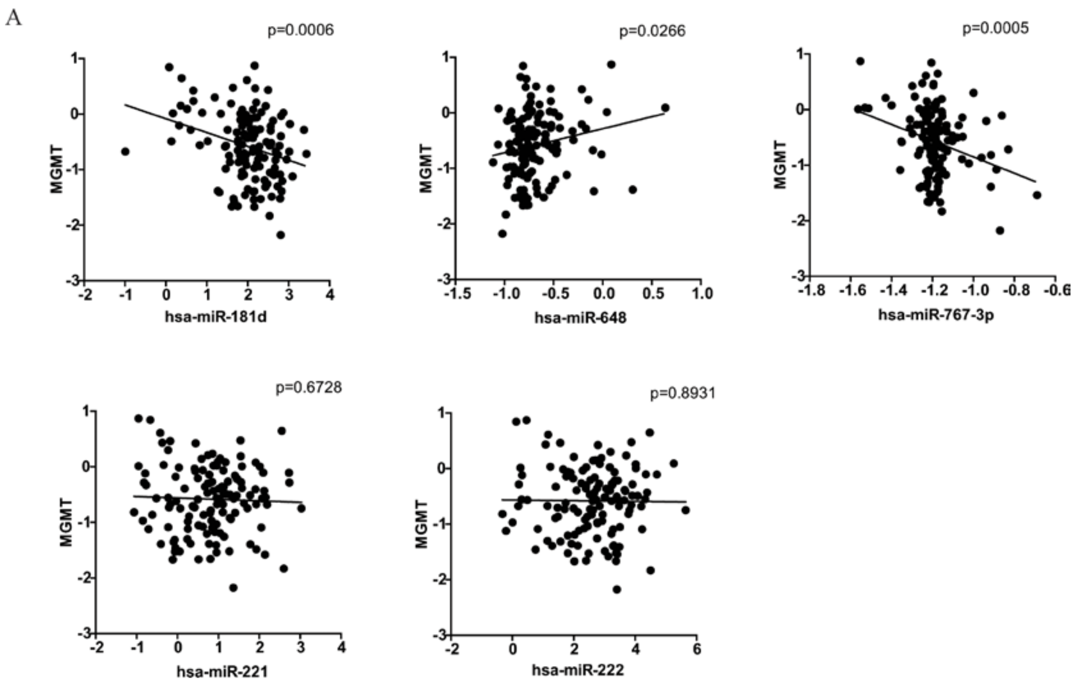

Supplementary Figure 3: Scatter plot demonstrating correlation between miRNA expression levels and MGMT expression levels in the TCGA. Glioblastoma specimens with unmethylated MGMT promoters (threshold cutoff < -0.25) were analyzed for correlation between MGMT and miRNA.

SuppTable-1:

miRNAs exhibiting strong MGMT silencing		
Number	miRNA name	% MGMT protein
1	hsa-miR-616-3p	36.70
2	hsa-miR-603	40.36
3	hsa-let-7f-2-3p	28.30
4	hsa-miR-497-3p	33.52
5	hsa-miR-155-5p	45.67
6	hsa-miR-767-3p	42.25
7	hsa-let7i-3p	30.32
8	hsa-miR-20a-3p	33.25
9	hsa-miR-612	44.82
10	has-miR-181b	35.7
11	hsa-miR-181d	37.21
12	hsa-let-7a2-3p	27.52
13	hsa-miR-30d	36.20
14	hsa-miR-589-3p	28.79
15	hsa-miR-429	46.84
16	hsa-miR-342-3p	48.76
17	hsa-miR-379	49.73
18	hsa-miR-548a-3p	36.76
19	hsa-miR-325	24.11
20	hsa-miR-361-5p	34.66
21	hsa-miR-338-5p	49.05
22	hsa-miR-30e-3p	19.79
23	hsa-miR-17	33.77
24	hsa-miR-191	33.47
25	hsa-miR-561	36.36
26	hsa-miR-371	41.14
27	hsa-miR-324-5p	45.46
28	hsa-miR-651	11.39

SuppTable-2:

miRNAs exhibiting MGMT silencing	
1	hsa-miR-18a-3p
2	hsa-miR-17-3p
3	hsa-miR-593-3p
4	hsa-miR-125b-2-3p
5	hsa-miR-539
6	hsa-miR-545-5p
7	hsa-miR-24-2-3p
8	hsa-miR-24-1-3p
9	hsa-miR-2054
10	hsa-miR-301a
11	hsa-miR-1975
12	hsa-miR-335-3p
13	hsa-miR-27b-3p
14	hsa-miR-154-3p
15	hsa-miR-130b
16	hsa-miR-146a-3p
17	hsa-miR-181c-3p
18	hsa-miR-1272
19	hsa-miR-124-3p
20	hsa-miR-508-5p
21	hsa-miR-371-3p
22	hsa-miR-548
23	hsa-miR-570
24	hsa-miR-491-3p
25	hsa-miR-1238
26	hsa-miR-578
27	hsa-miR-1270
28	hsa-miR-618
29	hsa-miR-329
30	hsa-miR-1244
31	hsa-miR-1255a
32	hsa-miR-29a-3p
33	hsa-miR-1206
34	hsa-miR-1278
35	hsa-miR-586
36	hsa-miR-483-3p
37	hsa-miR-545
38	hsa-miR-1323
39	hsa-miR-770-5p

40	hsa-miR-146b-5p
41	hsa-miR-875-5p
42	hsa-miR-10b
43	hsa-miR-532-5p
44	hsa-miR-377
45	hsa-miR-421
46	hsa-miR-208b
47	hsa-miR-367
48	hsa-miR-136
49	hsa-miR-1303
50	hsa-miR-580
51	hsa-miR-9
52	hsa-miR-7
53	hsa-miR-185
54	hsa-miR-449a
55	hsa-miR-30b
56	hsa-miR-30c
57	hsa-miR-153
58	hsa-miR-218
59	hsa-miR-96
60	hsa-miR-181a-3p
61	hsa-miR-22
62	hsa-miR-425
63	hsa-miR-369-5p
64	hsa-miR-323-5p
65	hsa-miR-208a
66	hsa-miR-93
67	hsa-miR-140-5p
68	hsa-miR-199a-5p
69	hsa-miR-877
70	hsa-miR-145
71	hsa-miR-33a
72	hsa-miR-29b
73	hsa-miR-205
74	hsa-miR-139-5p
75	hsa-miR-378
